# The *Rlm13* Gene, a New Player of *Brassica napus*–*Leptosphaeria maculans* Interaction Maps on Chromosome C03 in Canola

**DOI:** 10.3389/fpls.2021.654604

**Published:** 2021-05-12

**Authors:** Harsh Raman, Rosy Raman, Yu Qiu, Yuanyuan Zhang, Jacqueline Batley, Shengyi Liu

**Affiliations:** ^1^NSW Department of Primary Industries, Wagga Wagga Agricultural Institute, Wagga Wagga, NSW, Australia; ^2^Oil Crops Research Institute, Chinese Academy of Agricultural Sciences, Wuhan, China; ^3^School of Biological Sciences, University of Western Australia, Crawley, WA, Australia

**Keywords:** natural variation, resistance to *L. maculans*, canola, genome-wide analysis (GWA), linkage map construction

## Abstract

Canola exhibits an extensive genetic variation for resistance to blackleg disease, caused by the fungal pathogen *Leptosphaeria maculans*. Despite the identification of several *Avr* effectors and *R* (race-specific) genes, specific interactions between *Avr-R* genes are not yet fully understood in the *Brassica napus–L. maculans* pathosystem. In this study, we investigated the genetic basis of resistance in an F_2__:__3_ population derived from Australian canola varieties CB-Telfer (*Rlm4*)/ATR-Cobbler (*Rlm4*) using a single-spore isolate of *L. maculans*, PHW1223. A genetic linkage map of the CB-Telfer/ATR-Cobbler population was constructed using 7,932 genotyping-by-sequencing-based DArTseq markers and subsequently utilized for linkage and haplotype analyses. Genetic linkage between DArTseq markers and resistance to PHW1223 isolate was also validated using the *B. napus* 60K Illumina Infinium array. Our results revealed that a major locus for resistance, designated as *Rlm13*, maps on chromosome C03. To date, no *R* gene for resistance to blackleg has been reported on the C subgenome in *B. napus*. Twenty-four candidate *R* genes were predicted to reside within the quantitative trait locus (QTL) region. We further resequenced both the parental lines of the mapping population (CB-Telfer and ATR-Cobbler, > 80 × coverage) and identified several structural sequence variants in the form of single-nucleotide polymorphisms (SNPs), insertions/deletions (InDels), and presence/absence variations (PAVs) near *Rlm13*. Comparative mapping revealed that *Rlm13* is located within the homoeologous A03/C03 region in ancestral karyotype block “R” of *Brassicaceae*. Our results provide a “target” for further understanding the *Avr–Rlm13* gene interaction as well as a valuable tool for increasing resistance to blackleg in canola germplasm.

## Introduction

Blackleg, caused by the hemibiotrophic fungal pathogen *Leptosphaeria maculans* (Desmaz.) Ces. et de Not., is one of the highly widespread and devastating diseases of canola (*Brassica napus* L) and its relatives (Howlett, 2004; Rouxel and Balesdent, 2017). It continues to be a major threat to the sustainable production of canola across many parts of the world, particularly Australia, Europe, and North America (West et al., 2001; Fitt et al., 2006). Host resistance, qualitative (race-specific resistance mediated by *R* genes) and quantitative resistance [non-race-specific resistance mediated by quantitative trait loci (QTL)], is considered as the most effective and environmentally safe method of disease management. Since the 1970s, concerted efforts are being made in developing improved varieties of canola with resistance to *L. maculans* by accessing genetic variation from diploid and amphidiploid members of the *Brassicaceae* (Roy, 1984; Chèvre et al., 1997; Saal and Struss, 2005; Yu et al., 2012).

Qualitative resistance largely relies on the classical gene-to-gene hypothesis: avirulence/effector (*Avr*)–*R* gene recognition interaction (Flor, 1971), also called elicitor-triggered immunity (ETI, Jones and Dangl, 2006). In *Brassica* species, at least 18 genes, conferring “complete” resistance to blackleg, have been identified on the A_*r*_, A_*n*_, B_*n*_/B_*j*_, and C_*o*_ subgenomes of *Brassica* (Delourme et al., 2011; Raman et al., 2013, 2016a; Ferdous et al., 2019), while in *L. maculans*, at least eight *Avr* genes have been cloned (Gout et al., 2006; Fudal et al., 2007; Parlange et al., 2009; Ghanbarnia et al., 2012, 2018; Balesdent et al., 2013; Van de Wouw et al., 2014; Ghanbarnia et al., 2015; Plissonneau et al., 2016, 2018), which correspond to at least nine of the *R* genes (Ghanbarnia et al., 2018). To date, none of the blackleg *R* genes have been reported on the C_*n*_ subgenome of *B. napus*.

Both linkage and genome-wide association mapping approaches have been employed to identify loci having major and minor allelic effects for resistance to blackleg. Although *R* genes do not provide long-term disease control due to the adaptive nature of *L. maculans* (Rouxel et al., 2003; Li et al., 2006; Sprague et al., 2006), they are easy to manipulate in the breeding programs due to their simple (predominantly monogenic) inheritance and high additive genetic variance. Therefore, new sources of resistance are highly sought after by canola breeding programs to increase diversity in the resistance gene pool for commercial deployment into new varieties.

Understanding the genetic network involved in *Avr*–*R* gene interactions is crucial for the deployment of effective blackleg management strategies. However, investigating the genetic effects for race-specific *R* genes in field conditions, as well as in the ascospore shower test (with stubble collected from the field) under controlled conditions, is challenging due to the highly heterogenous nature of *L. maculans* populations, reliance on the environment (timing of spore release and weather conditions), method of evaluation, and genotype × environment interactions (Hayden et al., 2007). The *L. maculans*–*B. napus* system has also revealed several interesting *R–Avr* interactions, such as *AvrLm4-7/Rlm4* and *Rlm7* (Parlange et al., 2009) and *AvrLm1/Rlm1* and *LepR3* (Larkan et al., 2013). Recently, epistatic *R*–*Avr* interactions for *AvrLm3/Rlm3/AvrLm4-7* (Plissonneau et al., 2016) and *AvrLm5-9/Rlm9/AvrLm4-7* (Ghanbarnia et al., 2018) have also been reported. To gain an understanding of “novel” genes involved in resistance, we analyzed an intercross population derived from the cross, CB-Telfer (*Rlm4*)/ATR-Cobbler (*Rlm4*).

In this study, we identify a major locus, designated *Rlm13*, on chromosome C03 that accounts for genetic variation in resistance to *L. maculans* in an Australian canola population derived from the CB-Telfer/ATR-Cobbler. We further resequenced both the parental lines of the mapping population (CB-Telfer and ATR-Cobbler) and identified several structural sequence variants in the form of single-nucleotide polymorphisms (SNPs), insertions/deletions (InDels), and presence/absence variations (PAVs) near the *Rlm13*. Comparative mapping revealed that the *Rlm13* region is localized within the “R” ancestral block of *Brassicaceae*. Our results provide a novel “target” for further understanding *Avr*–*R* gene interactions, as well as a valuable tool for combining resistance loci in new varieties.

## Materials and Methods

### Plant Material

For linkage mapping, we developed an F_2_ population from a single F_1_ plant derived from the cross CB-Telfer/ATR-Cobbler. CB-Telfer is a current, homozygous Australian commercial variety, developed by Canola Breeders Western Australia Pty Ltd (CBWA, now NPZA, South Perth, Western Australia), while ATR-Cobbler (NMT040) is a current commercial canola variety developed by Nuseed Pty Ltd, Horsham, Victoria, Australia from the cross of ATR-Eyre/Ag-Emblem^1^. Both parental lines carry *R* genes for resistance to blackleg: CB-Telfer (*Rlm4*) and ATR-Cobbler [*Rlm4* and *Rlm9* (H; segregating for resistance)] (Marcroft et al., 2012).

### Phenotypic Evaluation for Resistance Isolates

To confirm specific *Avr*–*R* interaction between the parental lines of the mapping population, we utilized a differential set of single-spore isolates of *L. maculans*, which were previously characterized for avirulence (Marcroft et al., 2012). Isolates were procured from Marcroft Grains Pathology Pty Ltd, Horsham, Australia, and then multiplied at the Wagga Wagga Agricultural Institute (WWAI), NSW, Australia. Pycnidiospores were used for the cotyledon inoculation tests. The PHW1223 (*AvrLm5-6-8-9-S*) was the only isolate that showed a differential response between the parental lines (Supplementary Table 1); we used this isolate to phenotype F_2_ and F_2__:__3_ derived progenies from the CB-Telfer/ATR-Cobbler.

### Cotyledon Inoculation Assay

A total of 464 F_2_ lines, two parental lines, and five control cultivars [Westar (no *R* gene), AV-Garnet (*Rlm1* and *Rlm9*), Thunder TT (*Rlm4*), Charlton (*Rlm4*), and Caiman (*Rlm7*)] were evaluated for resistance to *L. maculans* under glasshouse conditions. The seedlings were grown in plastic trays (7 × 8 cells) in a controlled glasshouse at WWAI, Australia, and maintained at 20 ± 2°C. Each tray had a row of eight genotypes, representing either F_2_/parental or control cultivars. The cotyledon lobes of each plant were inoculated with the PHW1223 isolate, at a concentration of 10^7^ spores ml^–1^ as described previously (Raman et al., 2012). Briefly, 10-days-old plants were inoculated and then placed in a humidity chamber for 48 h at 100% relative humidity in the dark at 18°C to allow spore germination and further penetration into canola cells. Subsequently, inoculated plants were transferred to the glasshouse and maintained at 20 ± 2°C for 3–4 weeks. Disease symptoms on cotyledons were assessed at 18 days post inoculation, using a scale described previously (Koch et al., 1991): 0 (resistant response/no disease development) to 9 (highly susceptible/and profuse sporulation). Quantitative disease scores (0–9) were used to identify significant trait–marker associations for resistance. After phenotyping F_2_ lines for resistance, each line was transplanted individually into a small pot (70 mm square tubes, Garden City Plastics, Australia) and subsequently raised under glasshouse conditions.

Both parental lines, as well as each individual F_2_ plant from the CB-Telfer/ATR-Cobbler cross, were bagged at the flower bud initiation stage to generate selfed F_2__:__3_ families. To confirm the disease scores of F_2_ plants, 96 F_2__:__3_ families were randomly selected and assessed for response to infection using a cotyledon assay with the PHW1223 isolate. Eight seedlings per F_2__:__3_ derived family were grown in plastic trays (7 × 8 cells), accommodating seven accessions in each tray. Phenotyping and assessment for resistance to *L. maculans* were carried-out as described above.

### DNA Isolation and Genotyping

DNA was isolated from young leaves from the same parental and F_2_ lines that were evaluated for resistance to PHW1223 using a standard cetyltrimethylammonium bromide (CTAB) method. DNA samples were genotyped with DArTseq (Raman et al., 2014) and 60K Illumina Infinium SNP markers (Clarke et al., 2016). SNP genotypes generated from the 60K Illumina Infinium were scored using the Genotyping Module of Genome Studio Data Analysis software (Illumina Inc., San Diego, United States). Polymorphic DArTseq SNPs and presence/absence markers were determined by aligning the Illumina reads with the sequenced genomes of *B. napus* cv. Darmor-*bzh* version 4.1 (Chalhoub et al., 2014), after trimming the barcodes and using Bowtie version 0.12 as described previously (Raman et al., 2016b). Markers having ≥ 90% call rate and < 10% missing data were used for linkage analysis.

In order to confirm the presence of *Rlm9* in the population, all individuals were genotyped using *Rlm9*-specific primers (Rlm9F1: 5′-TCGTATAGTTCTTATCGCCTGCC-3′, Rlm9R1: 5′-TCCGTAAGTCAGGCTATAGTGT-3′). Amplification was performed using DreamTaq Green PCR Master Mix (2X, Thermo Fisher Scientific) with the reaction mixture prepared following the manufacturer’s protocol. The cycling conditions were initial denaturation at 95°C for 3 min, 35 cycles of 95°C for 30 s, 60°C for 30 s, and 72°C for 45 s, with a final elongation of 5 min. PCR products were visualized on an agarose gel.

### Statistical and Linkage Analysis

A set of 7,932 DArTseq markers that showed segregation among DH lines were selected for genetic linkage map construction, as detailed by Raman et al. (2016b). Map distance in centimorgan (cM) was calculated using the Kosambi mapping function. A genome scan was performed to identify any associations for resistance to blackleg using the linear marker analysis algorithm, implemented in the SVS package^2^. The allele frequency of SNP markers and their association with mean disease scores (from F_2_ lines) were determined by using linear marker regression, *F*-test, and correlation/trend test, implemented in SVS. For reducing Type 1 and Type II errors, multiple testing corrections for Bonferroni adjustment (on SNPs), false discovery rate, and full genome scan permutations (5,000 iterations) were conducted in the SVS package. We further performed haplotype association analysis using the expectation/maximization (EM) method following 1,000 iterations, a dynamic window size of 5 kb, and an EM convergence tolerance value of 0.0001. Trait–marker association was graphically represented using the MapChart software (Voorrips, 2002).

### Physical Mapping of *Rlm13*

To determine the position of the *Rlm13* locus in relation to the previously reported QTL for resistance to blackleg (Raman et al., 2016a, 2018), we performed comparative mapping of marker sequences underlying QTL using the Darmor-*bzh* reference genome of *B. napus* version 4.1 (Chalhoub et al., 2014). If the physical positions of the genomic region (QTL interval) and *Rlm13* overlapped (within 50 kb) on C03, the genomic region was considered to be the same. Since the *LepR4* locus for resistance to blackleg is mapped on chromosome A06 (Yu et al., 2013) and the latter showed synteny with the homoeologous chromosomes A03/C03, we searched the sequences of marker intervals encompassing *LepR4*.

### Putative Candidate Genes for *Rlm13*-Mediated Resistance

The proximity of candidate genes to identified associations was inferred based on the functional annotation of the *Arabidopsis thaliana* genome and implemented in the reference sequenced genome of “Darmor-*bzh*.” Sequences were searched for their identities with the disease resistance NLR-*R* genes (Chalhoub et al., 2014; Alamery et al., 2018; Tirnaz et al., 2020) using the RGAugury function using the *B. napus* genome^3^ to obtain their physical map positions.

### Resequencing of Parental Lines

Whole-genome resequencing (2 × 150 bp) of both parental lines, CB-Telfer and ATR-Cobbler, was performed at the Novogene facility (Novogene Co., Ltd, Hong Kong) using the Illumina HiSeq 2000 sequencing platform. The coverage of the parental lines ranged from 83.7 × (94.6 Gb, ATR-Cobbler) to 90.1 × (102.6 Gb, CB-Telfer). Reads were mapped onto the “Darmor-*bzh*” reference assembly (version 4.1) using BWA version 0.7.8 (Li and Durbin, 2010). SNP and InDel calling based on the short-read alignment data were performed using the GATK haplotype caller (version 3.5).

Genomic structural variation (SV) detection for two parental lines was performed using novoBreak (Chong et al., 2017) compared against the Darmor-*bzh* reference sequence (version 4.1). SVs inferred by no less than 10 reads were further filtered with the following conditions: (1) more than five supporting split reads or (2) no fewer than three discordant read pairs. We detected deletions, duplications, and inversions with sizes between 100 bp and 1 Mb. Two adjacent SVs were identified as the same SV if their start and end positions varied no more than 1 kb, and the overlapping region was more than 50% of the total size of that two SVs.

### Identification of Homoeologous Exchange (HE)

To identify the HE events between A_*n*_ and C_*n*_ subgenomes in each of the two parental lines, we followed a standard protocol, described earlier with minor parameter optimization (Chalhoub et al., 2014). First, the resequenced reads for each line were aligned to the Darmor-*bzh* reference genome version 4.1 by using BWA with default parameters (Li and Durbin, 2010). Second, successive 10-kb windows are scanned to calculate the average depth on the whole genome. If the coverage depth is more than 1.5-fold of the average coverage of the whole genome, this was defined as a double coverage. By contrast, if the coverage depth is less than half of the average coverage of the whole genome, it would be defined as a deletion coverage. Adjacent windows with depths greater than the threshold and that were at most 10 windows distant were linked together. Only the regions spanning more than 10 windows (100 kb total) were retained as HE regions. Finally, the read coverage depth was used to indicate HE, where regions with double-read coverage were considered duplicated while regions with low or no coverage in its corresponding syntenic region of homoeologous subgenomes were considered to be deleted/replaced.

### Comparative Mapping of *Rlm13* Associated Markers to Ancestral Blocks of *Brassicaceae*

We further sought to identify whether marker intervals associated with *Rlm13* in CB-Telfer/ATR-Cobbler are located within the same duplicated regions on chromosome A03/C03 for resistance to *L. maculans* that were detected previously under field and glasshouse conditions (Fomeju et al., 2014; Fopa Fomeju et al., 2015; Raman et al., 2016a, 2018; Kumar et al., 2018). The marker sequences associated with *Rlm13* were searched for their identities using the reference *B. napus* cv. Darmor-*bzh* and ancestral karyotype blocks of *A. thaliana* (TAIR10^4^), which is considered as the pre-triplication ancestor of *B. napus*. The co-location between NLR gene/marker sequences underlying QTL/synteny (AK) and physical positions of *Rlm13* were analyzed using Microsoft Excel.

## Results

### A Single Race-Specific Gene Controls Resistance to *L. maculans*

To confirm the specific *Avr*–*R* interactions in parental lines of a mapping population, CB-Telfer (*Rlm4*) and ATR-Cobbler (*Rlm4*, heterogenous), we utilized a differential set of *L. maculans* isolates. The PHW1223 (*AvrLm5-6-8-9-S*) was the only isolate that revealed a contrasting disease response between parents (Supplementary Table 1). Inferred *R* gene profiles from a differential set of isolates suggested that ATR-Cobbler likely possesses *Rlm9*, consistent with earlier findings (Marcroft et al 2012). Recently, the *Rlm9* gene (BnaA07g20220D), encoding the wall-associated kinase-like (WAKL) protein, has been cloned in *B. napus* (Larkan et al., 2020); we resequenced this gene from both the parental lines CB-Telfer and ATR-Cobbler and compared it with the reference Darmor-*bzh* genome sequence version 4.1 (Supplementary Table 2). There were three identical SNPs in exon 1 at the coordinates 15,912,895 bp (C > T), 15,912,904 bp (T > C), and 15,912,927 bp (C > A) in both parental lines compared to the Darmor, which harbors *Rlm9* (Delourme et al., 2004). Sequence analysis suggests that the *Rlm9* allele present in the parental lines does not contribute to variation in resistance in this mapping population. Both parents contained a susceptible allele, along with all progeny when tested with *Rlm9* gene-specific primers.

**FIGURE 1:**
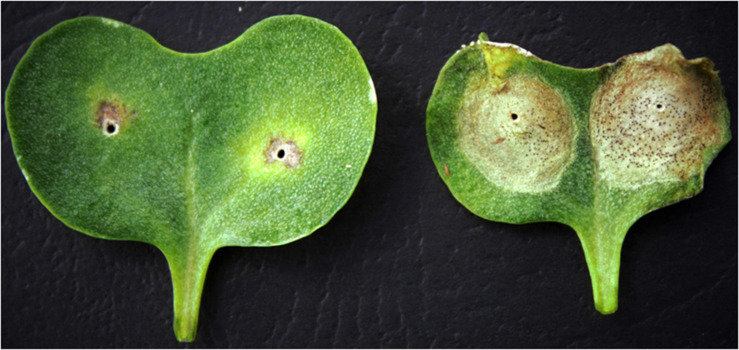
Infection response of parental lines CB-Telfer (maternal, *Rlm4*) and ATR-Cobbler (paternal, *Rlm4* and *Rlm9*) to the *L. maculans* isolate PHW1223 (*AvrLm5-6-8-9-S*). The visible symptoms of blackleg commenced after 7–10 days of inoculation. Resistant parental and intercross lines displayed a limited cotyledon lesion compared to susceptible lines, which displayed extensive cotyledon lesions, characterized by discoloration and pyncindia sporulation (after 21 days of inoculation).

**FIGURE 2:**
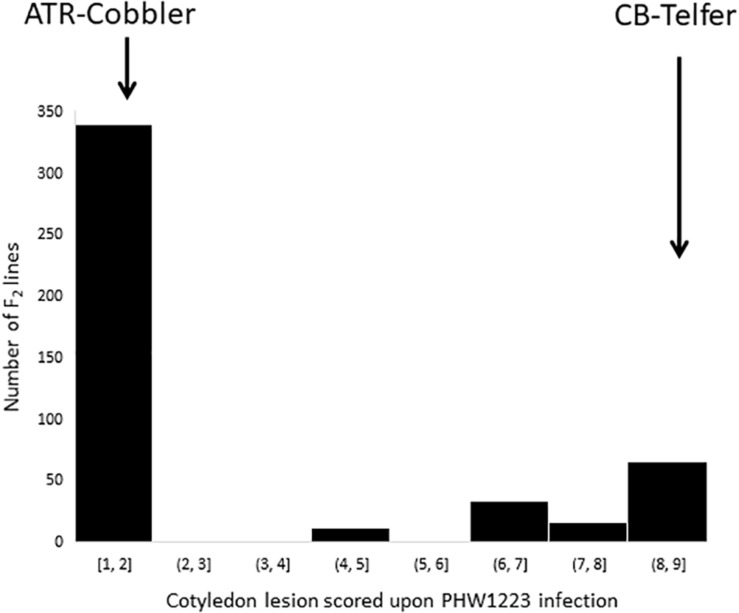
Frequency distributions of the cotyledon lesion scores in an F_2_ population derived from the CB-Telfer/ATR-Cobbler. Plants were infected with a single-spore isolate, PHW1223, at the cotyledon stage. Parental means are shown with arrows.

To understand the genetic basis underlying the differential disease expression response to the PHW1223 isolate (Supplementary Table 1), we phenotyped a total of 464 F_2_ lines from CB-Telfer/ATR-Cobbler. Upon infection, ATR-Cobbler displayed a low cotyledon lesion score (1) for resistance, whereas the CB-Telfer had a high disease lesion score of (8) for susceptibility (Figure 1). The cotyledons of resistant genotypes exhibited a classical hypersensitive response with limited necrosis but no sporulation, while the susceptible parental lines showed extensive cell death and sporulation. Phenotypic evaluation of 464 F_2_ plants exhibited a bimodal distribution of disease scores to isolate PHW1223 (Figure 2). A Chi-square test (χ^2^ = 0.74; *P*_0_._05_ = 0.39) confirmed that a single locus controls resistance in this F_2_ population, as the allelic ratio conforms to a 3 (340 resistant plants)-to-1 (116 susceptible plants) segregation. The observed response is consistent with the highly specific interaction of a pathogen effector (*Avr* protein) and host resistance (*R*) protein, for the gene-for-gene complementarity hypothesis (Flor, 1971; Keen, 1990; Jones and Dangl, 2006). In the F_2_ population, homozygous resistant genotypes could not be discriminated conclusively, based on the extent of cotyledon infection, from heterozygous resistant ones, while those which lacked a resistance allele developed large lesions on the inoculated cotyledons. To verify the disease scores of each individual F_2_ line, we randomly selected 96 lines from 464 F_3_ families. Of these, 30 were homozygous resistant, 42 were heterozygous (segregation for both resistance and susceptibility), and 24 were homozygous susceptible. No segregation was observed within susceptible F_3_ families, suggesting that phenotypic conditions for disease expression were optimal. The data on F_2__:__3_ families fitted in a codominant monogenic segregation ratio (χ^2^ = 2.25, *P*_0_._05_ = 0.32), indicating that a single gene controls variation in resistance to isolate PHW1223.

### Construction of Linkage Map

To locate the locus for race-specific resistance in ATR-Cobbler, we constructed a linkage map of the genetic population derived from CB-Telfer/ATR-Cobbler. A total of 7,932 polymorphic DArTseq markers that showed segregation in the F_2_ population were utilized for map construction. These markers were grouped into 21 linkage groups, representing the 19 haploid canola chromosomes and unassigned ChrA_random and ChrC_random linkage groups (Table 1). The total length of the genetic map was 2,252.22 cM, with an average coverage of one marker per 0.28 cM. Of the 19 chromosomes of *B. napus*, A06 had the highest marker density (4.42/cM), while chromosome C01 had the lowest marker coverage and C05 had the lowest marker density (2.37/cM). The majority of the DArTseq markers (75%) could be anchored on the reference “Darmor-*bzh*” genome (Supplementary Table 3).

**TABLE 1:** Linkage map of the CB-Telfer/Ag-Cobbler population constructed using DArTseq markers.

Linkage group	Markers mapped	Linkage length (cM)	Marker density/cM	Marker coverage
A01	494	143.33	3.45	0.29
A02	260	78.82	3.3	0.3
A03	397	106.14	3.74	0.27
A04	421	122.96	3.42	0.29
A05	341	105.88	3.22	0.31
A06	864	195.66	4.42	0.23
A07	260	84.09	3.09	0.32
A08	325	88.92	3.66	0.27
A09	458	126.86	3.61	0.28
A10	452	112.5	4.02	0.25
**Subtotal A subgenome**	4,272	1165.16	3.67	0.27
C01	45	16.86	2.67	0.37
C02	559	161.77	3.46	0.29
C03	833	194.12	4.29	0.23
C04	648	187.44	3.46	0.29
C05	79	33.31	2.37	0.42
C06	353	97.59	3.62	0.28
C07	492	139.17	3.54	0.28
C08	306	91.44	3.35	0.3
C09	262	95.73	2.74	0.37
Cnn_random_1	59	54.18	1.09	0.92
Cnn_random_2	24	15.44	1.55	0.64
**Total C subgenome**	3,660	1087.06	3.37	0.3
**Total A + C subgenome genomes**	7,932	2252.22	3.52	0.28

### Genetic Mapping of *Rlm13*

**FIGURE 3:**
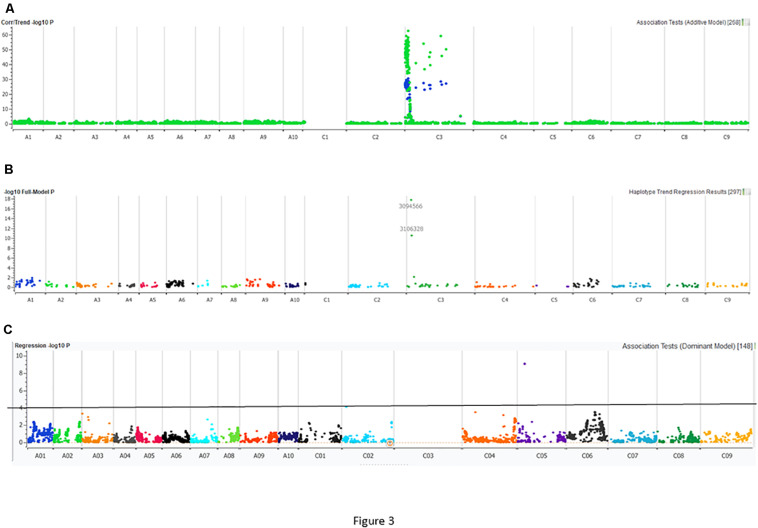
Manhattan plots describing marker associations for resistance to the PHW1223 isolate of *L. maculans* in an intercross population derived from CB-Telfer/ATR-Cobbler. Resistance to *L. maculans* was assessed at the cotyledon stage under glasshouse conditions. **(A)** Markers highlighted in *green* represent associations detected for resistance to the PHW1223 isolate using linear marker regression (additive model), and those in *blue* represent associations detected for resistance to the PHW1223 isolate using correlation/trend (additive model). **(B)** Marker associations identified for resistance using haplotype trend regression. **(C)** Marker association identified for resistance to PHW1223 isolates (without markers mapped on chromosome C03). Different colors represent chromosomes of *B. napus* (A01–A10 and C01–C09). Significant associations with a −log_10_ *P* ≤ 3 are shown with a solid horizontal line (in black).

**FIGURE 4:**
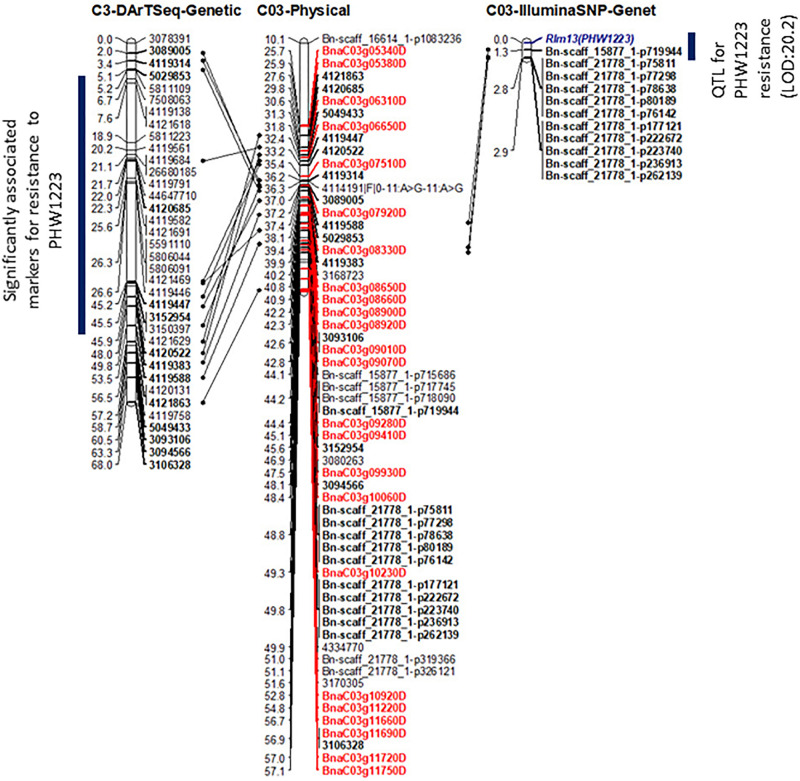
Genetic and physical mapping of the *Rlm13* locus in relation to DArTseq and Illumina SNP-based markers. The order of markers was determined using the Record program. The marker order is shown on the *right-hand side*, while the genetic and physical positions of markers and candidate genes (labeled in Red color) are on the *left-hand side*. Positions of common markers across genetic and physical maps are in bold. For clarity, physical distances are given in 0.1 Mb.

Utilizing the linkage map and cotyledon lesion score data, we scanned the genome for association with resistance to the PHW1223 isolate using multiple association tests (Table 2). We dropped markers with a call rate < 0.9 and selected a subset of 6,849 markers for trait–marker analyses. On chromosome C03, a major QTL for resistance was identified (Figure 3 and Table 2). Regression analysis showed that the paternal parent, ATR-Cobbler, contributed the allele for resistance, consistent with the response to PHW1223. We further confirmed the linkage between DArTseq markers and resistance to PHW1223 using 60K Illumina SNP markers, genotyped on selected F_2_ plants exhibiting resistance and susceptibility to the PHW1223 isolate. A genome scan using linear marker regression revealed that an Illumina SNP marker, Bn-scaff_15877_1-p719944, on chromosome C03 showed the most significant association (−log_1__0_ *P* = 20.22), followed by Bn-scaff_21778_1-p75811 (−log_1__0_ *P* = 13.35) (Supplementary Table 4). The allelic effect confirmed that resistance was contributed by the resistant parent, ATR-Cobbler, as observed with DArTseq marker analysis. Haplotyping-based association analysis also identified significant association with resistance to PHW1223 (−log_1__0_ *P* = 17.79) with the marker haplotype “3094566-3087181-4334770” mapped at 4.87 Mb on the Darmor-*bzh* genome. Since all the associated markers on chromosome C03 had high logarithm of the odds (LOD) scores in multiple tests (Table 2 and Supplementary Table 4), we binned the disease scores into discrete categories (resistance and susceptibility). Linkage analysis showed that the resistance to isolate PHW1223 indeed maps on chromosome C03 and was 1.3 cM from the Bn-scaff_15877_1-p719944 Illumina SNP marker (Figure 4). So far, no race-specific gene for resistance (*R*)/*Avr* interaction on C03 chromosome of *B. napus* has been reported in the literature; as such, we designated this locus for race-specific resistance to *L. maculans* as a novel locus, *Rlm13*.

**TABLE 2:** Genetic association between GBS-based DArTSeq markers and resistance to *L. maculans*. Only highly significantly associated markers are shown herein.

Method	Marker/haplotype	Chromosomal location	Position on the Darmor-*bzh* assembly (bp)	LOD (−log^10^ *P*)
Linear marker regression	5591110	C03	Unknown	69.87
	4119314	C03	3,622,355	62.60
*F*-test (dominant model)	5591110	C03	Unknown	69.87
	4119314	C03	3,622,355	62.60
Correlation/Trend test	5591110	C03	Unknown	31.29
(dominant model)	4119314	C03	3,622,355	30.39
Fisher’s exact test	5591110	C03	Unknown	35.46
	4119314	C03	3,622,355	34.73
Odd ratio	7508063	C03	Unknown	562.5 (R vs. S)
	5049433	C03	3,125,686	(412 R vs. S)
Haplotyping analysis (Chi square)	4120432, 4119383 (AA)	C03	3,849,507	57.14
Haplotyping analysis (Odd ratio)	3093106, 4109656, 3155140 (AAA)	C03	4,257,471	267.14 (R vs. S)

*R: Resistant, S: Susceptible to PHW1223 isolate. *For details, see Supplementary Table 4.**

### Physical Mapping of *Rlm13*

To determine the physical location of *Rlm13*, we compared the sequence identities between the significant (DArTseq and Illumina SNP) marker sequences and the Darmor-*bzh* reference sequence. All the top 50 DArTseq markers that were significantly associated with *Rlm13* were located on chromosome C03, with the majority of them located within the 3.12–6.8 Mb interval (Figure 4 and Supplementary Table 4). Several Illumina SNP markers that had significant association with resistance were also mapped within the 4.41–5.7 Mb interval (Supplementary Table 4). Of these, the Bn-scaff_15877_1-p719944 marker, located on chromosome C03 at 4,417,287 bp (Clarke et al., 2016), had the highest LOD score (20.2). A further six Illumina SNP markers having a −log_1__0_ *P* score ≤ 13 were also located within 0.5 Mb from the Bn-scaff_15877_1-p719944 marker (Figure 4 and Supplementary Table 3). Mapping of multiple markers that revealed significant association with resistance to isolate PHW1223 within a small genomic region on C03 suggests that the *Rlm13* is indeed localized in this region.

### Localization of *Rlm13* in Relation to Known Resistance Loci

We compared the physical positions of significantly associated markers with *Rlm13* and previously identified QTL for resistance, which was assessed previously under (i) glasshouse conditions with isolate PHW1223 (used in this study) and five isolates representing pathogenicity group 4, (ii) an ascospore shower test in diverse Australian *B. napus* germplasm (Raman et al., 2016a), and (iii) natural field conditions in diverse European and Australian *B. napus* germplasms (Fomeju et al., 2014; Fopa Fomeju et al., 2015; Jestin et al., 2015; Rahman et al., 2016; Kumar et al., 2018) on chromosomes A03 and C03. This analysis showed that the *Rlm13* region does not overlap with QTL intervals for quantitative resistance on C03 described in a diverse panel of accessions, representing winter, spring, semi-winter, and rutabaga types (Fopa Fomeju et al., 2015; Rahman et al., 2016). However, *Rlm13* is located within the QTL regions for quantitative resistance, detected on C03 in the Darmor/Bristol DH population and genome-wide association study (GWAS) panel of *B. napus* (Fomeju et al., 2014; Jestin et al., 2015; Raman et al., 2016a; Kumar et al., 2018). The *Rlm13* was located within the QTL region, spanning 3.62–4.25 Mb of the Darmor-*bzh* genome for resistance identified previously in a GWAS panel of *B. napus* accessions to six single-spore isolates of *L. maculans*, D1, D2, D6, D9, D10 (PHW1223), and 04MGPS021 (Raman et al., 2016a), suggesting that it may be conditioning resistance response to *L. maculans* across multiple isolates; some of the associations were detected within 4 kb (Supplementary Table 5).

Earlier studies have shown that the race-specific resistance locus *LepR4* maps on chromosome A06 in *B. napus* (Yu et al., 2013), which shares homoeology with group 3 chromosomes (Chalhoub et al., 2014). Therefore, we determined whether the genomic regions associated with the *Rlm13* and *LepR4* resistance are present on the same homoeologous genomic regions. First, we investigated the syntenic relationship among marker intervals, collinear with *Brassica rapa* genes on A06, flanking *LepR4* (Bra017959, Bra018037, Bra018198, and Bra024309, Yu et al., 2013), *Brassica oleracea*, and *B. napus* genomes. These *B. rapa* genes on A06 showed synteny with *B. oleracea* genes Bo3g132890 and Bo3g101190 located on chromosome O3 and Bo8g078980 and Bo8g077270 on O8 and *B. napus* genes BnaA06g15600D and BnaA06g23540D on A06, BnaC03g56070D on C03, and BnaC08g20450D and BnaC08g21330D on C08 (Supplementary Table 6). Of these, BnaC03g56070D was the only one mapped on C03 of the Darmor-*bzh* genome, but at 45,361,849–45,362,475 bp, suggesting that the *LepR4* region does not map near *Rlm13*.

**FIGURE 5:**
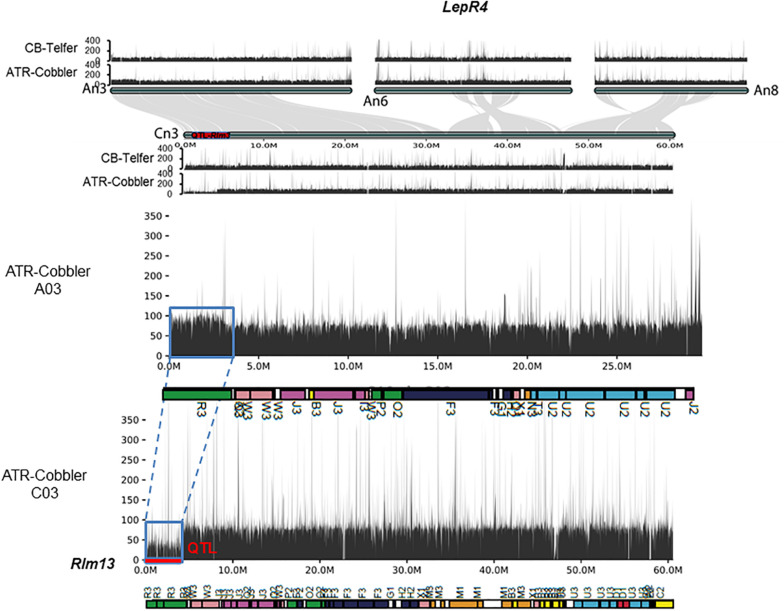
Syntenic relationships between *LepR4* (on A06) and homoeologous group 3 chromosomes, encompassing the *Rlm13* region on chromosome C03. The gene syntenies between A03, A06, A08, and C08 are shown with gray connections. The *Rlm13* region is marked in red. The coverage depth of homoeologous chromosomes was calculated after mapping resequenced reads to the Darmor-*bzh* genome assembly version 4.1. In ATR-Cobbler, the coverage depth of the *Rlm13* region on chromosome C03 is less than 50 (1×), while in A03, the coverage depth is double (∼100), indicating the occurrence of HE between A03 and C03. The syntenic region, subjected to HE, is marked with dotted/solid lines (in blue). Gene sequences which were located in different ancestral blocks are also marked in different colors. Localization of marker sequences to ancestral blocks was carried out using the method described previously (Zou et al., 2016).

We further aligned the genomic sequences of both parental lines with > 80 × Illumina read coverage and interrogated the syntenic regions on the A_*n*_ and C_*n*_ subgenomes of *B. napus*, corresponding to *LepR4*. As expected, chromosome C03 exhibited extensive synteny to genomic regions on chromosomes A03, A06, and A08 (Figure 5). Our results reconfirmed that the 3.62–4.25 Mb genomic region encompassing *Rlm13* is not syntenic to the *LepR4* region on A06. These findings indicate that *Rlm13* indeed represents a new locus for resistance and is different to *LepR4* derived from *B. rapa* ssp. *sylvestris* (Yu et al., 2013).

### SV Between Parental Lines

**FIGURE 6:**
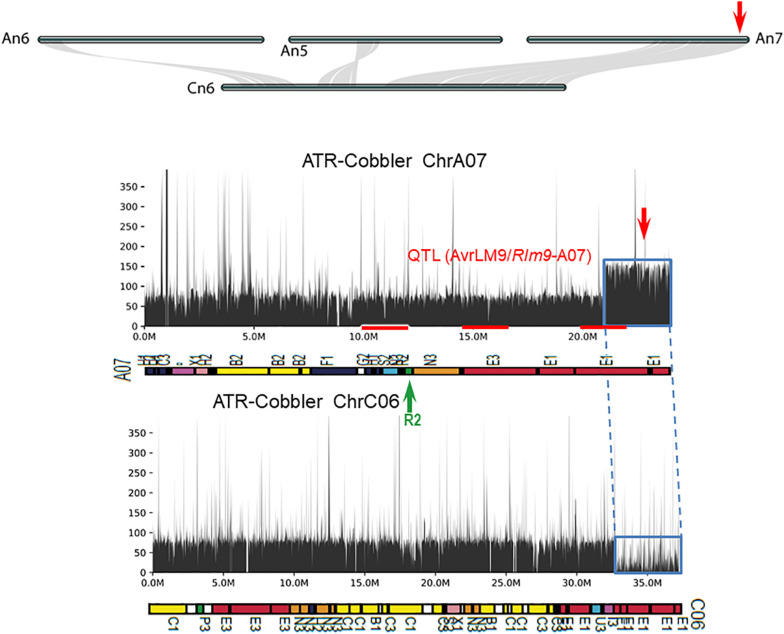
Syntenic relationships between homoeologous chromosome harboring *LepR4* and *Rlm9* genes for resistance to *L. maculans*. The gene syntenies between A05, A06, A07, and C06 are shown with gray connections. The *Rlm9* region maps to 15.1 Mb of the Darmor-*bzh* sequence and is marked in red. The coverage depth of homoeologous chromosomes was calculated after mapping resequenced reads to the Darmor-*bzh* genome assembly version 4.1. Gene syntenies show that in ATR-Cobbler, the coverage depth of the C06 region is almost nil, while in A07, the coverage depth is more than double, illustrating the occurrence of HE between A07 and C06. The syntenic region, subjected to HE, is marked with dotted/solid lines (in blue). Gene sequences which were located in different ancestral blocks are also marked in different colors. Localization of marker sequences to ancestral blocks was carried out using the method described previously (Zou et al., 2016).

Using the resequenced data of CB-Telfer and ATR-Cobbler, we identified several SNPs, InDels, and SVs in the form of transversion, deletion, duplication, and inversions, which were polymorphic between the parental lines, CB-Telfer and ATR-Cobbler, of a mapping population. To understand whether the *Rlm13* region is subjected to HE, we used the read coverage depth of resequenced genomes of CB-Telfer and ATR-Cobbler. HE analysis revealed that the A03 region corresponding to *Rlm13* on chromosome C03 is in fact subjected to a non-reciprocal duplication (Figure 5). We also found that chromosome A07 is also duplicated, as the corresponding read coverage depth in the C06 region is almost half (Figure 6). However, this duplicated region on chromosome A07 does not represent the *Rlm9* genomic region (15.9 Mb, Raman et al., 2018; Larkan et al., 2020), which was anticipated to map in the intercross population from CB-Telfer (*Rlm4*)/ATR-Cobbler (*Rlm4* and *Rlm9*). Both these non-reciprocal HEs (C03 to A03 and C06 to A07) occurred from the C_*n*_ subgenome to A_*n*_ subgenome in the CB-Telfer/ATR-Cobbler. Interestingly, A03/C03 HE occurred at the start of the chromosomes, while the A07/C06 occurred at the end of the homoeologous chromosomes (Figures 5, 6). The SVs are known to occur in *B. napus* and play an important role in phenotypic and functional diversity in various plants which have undergone genome duplication and fractionation events (Chalhoub et al., 2014; Clark and Donoghue, 2018; Hurgobin et al., 2018). Research to establish the role of HE and PAVs in resistance to *L. maculans* is in progress.

### Collinearity of *Rlm13* With Ancestral Blocks of *Brassicaceae* and Identification of Candidate Genes for Resistance to *L. maculans*

To investigate whether *Rlm13* is located in the duplicated homoeologous regions involved in resistance to blackleg, we searched the synteny between significantly associated markers with *Rlm13* and 22 ancestral blocks of *Brassicaceae*, as investigated in earlier studies (Schranz et al., 2006; Delourme et al., 2013; Fomeju et al., 2014; Parkin et al., 2014; Fopa Fomeju et al., 2015). Comparative mapping showed that the *Rlm13* region is collinear to sequences of A02, A03, A10, C02, and C09, as shown previously (Fopa Fomeju et al., 2015). The syntenic region of *Rlm13* corresponded to the most fractionated subgenome MF2 of the R block, delimited with coordinates of the Darmor-*bzh*, 3,138,662–5,148,532 bp on C03 (Fopa Fomeju et al., 2015). However, this study did not detect any significant SNP association for resistance to blackleg in R and W blocks of chromosome C03 (Supplementary Table 7).

We further searched candidate genes within a 0.5-Mb window either side of the physical location of the *Rlm13* region. Plausible candidates for disease resistance were selected, which are annotated in the Darmor-*bzh* genome (Chalhoub et al., 2014; Alamery et al., 2018; Tirnaz et al., 2020). At least 24 *R* genes were identified in the *Rlm13* region (Figure 4 and Table 3). Of these, 10 were RLKs, four were RLPs, seven were NBS-LRRs, and three were TM-CC. So far, three *R* genes (two RLP-type genes: *LepR3* and *Rlm2* and one *RLK* gene: *Rlm9*) were identified for resistance to *L. maculans* (Larkan et al., 2013, 2015, 2020). The candidates for *Rlm13* genes were located within 0.4 Mb of the Darmor-*bzh* genome and were localized in the R ancestral block in the most fractionated (MF2) subgenome on chromosome C03 (Supplementary Table 7). The BnaC03g12220D (DANGEROUS MIX2H gene) was located in the W block in the subgenome MF2. Of the plausible candidate genes, at least nine of them showed InDel (11), SNP (163), and SVs (21) in the form of transversion, inversion, duplication, and deletion between CB-Telfer and ATR-Cobbler (Supplementary Table 2), which remains to be validated for their association to *Rlm13*-mediated resistance.

**TABLE 3:** Candidate genes identified in the vicinity of the *Rlm13* genomic region associated with resistance to *L. maculans*, causing blackleg disease in canola (*B. napus* L.).

*B. napus* gene ID	Chromosome	Start (bp)	End (bp)	Length (bp)	*R* gene Type	Motif
BnaC03g05340D	C03	2,573,230	2,575,365	2,135	RLK	Other_receptor
BnaC03g05380D	C03	2,592,592	2,596,564	3,972	NBS-encoding	TNL
BnaC03g06310D	C03	3,057,621	3,060,722	3,101	RLK	LRR
BnaC03g06650D	C03	3,183,193	3,185,758	2,565	TM-CC	TM-CC
BnaC03g07510D	C03	3,539,026	3,542,690	3,664	RLK	LRR
BnaC03g07920D	C03	3,723,953	3,729,620	5,667	RLP	LRR
BnaC03g08330D	C03	3,939,605	3,942,943	3,338	RLP	LRR
BnaC03g08650D	C03	4,082,610	4,087,660	5,050	NBS-encoding	TNL
BnaC03g08660D	C03	4,089,063	4,094,327	5,264	NBS-encoding	TNL
BnaC03g08900D	C03	4,222,525	4,224,460	1,935	NBS-encoding	NBS
BnaC03g08920D	C03	4,227,171	4,232,582	5,411	NBS-encoding	TN
BnaC03g09010D	C03	4,264,274	4,265,687	1,413	NBS-encoding	TX
BnaC03g09070D	C03	4,282,511	4,286,710	4,199	RLK	Other_receptor
BnaC03g09280D	C03	4,438,959	4,443,152	4,193	RLP	LRR
BnaC03g09410D	C03	4,510,238	4,510,891	653	NBS-encoding	TX
BnaC03g09930D	C03	4,750,546	4,751,916	1,370	RLK	Other_receptor
BnaC03g10060D	C03	4,843,570	4,844,160	590	TM-CC	TM-CC
BnaC03g10230D	C03	4,934,330	4,938,969	4,639	TM-CC	TM-CC
BnaC03g10920D	C03	5,277,709	5,279,359	1,650	RLP	LRR
BnaC03g11220D	C03	5,480,793	5,482,813	2,020	RLK	Other_receptor
BnaC03g11660D	C03	5,669,051	5,680,319	11,268	RLK	LRR
BnaC03g11690D	C03	5,686,484	5,689,755	3,271	RLK	LRR
BnaC03g11720D	C03	5,699,183	5,701,757	2,574	RLK	LRR
BnaC03g11750D	C03	5,705,260	5,711,418	6,158	RLK	LRR

## Discussion

In this study, we identified a new locus for resistance, *Rlm13*, on chromosome C03 that confers resistance to the PHW1223 isolate of *L. maculans* in an Australian canola population derived from CB-Telfer/ATR-Cobbler. Previously, several genes for qualitative resistance to *L. maculans* were mapped in *B. napus*, such as *Rlm1*, *Rlm3*, *Rlm4*, *Rlm7*, and *Rlm9* on A07 (Dion et al., 1995; Mayerhofer et al., 1997; Delourme et al., 2004; Tollenaere et al., 2012; Raman et al., 2013); *Rlm2*, *BlmR2*, and *LepR3* on chromosome A10 (Delourme et al., 2004; Long et al., 2011; Larkan et al., 2013, 2015); *Rlm12* on chromosome A01 (Raman et al., 2016a); *LepR1* and *LepR2* on A02 (Yu et al., 2005); and *LepR4* on A06 (Yu et al., 2013). To date, no *R* gene for resistance on chromosome C03 has been reported in *B. napus* and its ancestral species (A03 in *B. rapa* and C03 in *B. oleracea*).

PHW1223 has *AvrLm5-6-8-9-S* genes for avirulence, which interact with the corresponding *R* genes in a host (Delourme et al., 2004; Raman et al., 2018); however, we could not identify any association on chromosomes A02, A07, A06, and A10 for resistance with known *R* genes (described above). We further checked whether identification of the *Rlm13* locus is due to the masking effect of *Rlm1*, *Rlm3*, *Rlm4*, *Rlm7*, and *Rlm9* genes; this was discounted by “hiding” markers on chromosome C03. Interestingly, we did not detect any significant association with resistance to the PHW1223 isolate on chromosome A07 (Figure 2). These results implicate that identification of *Rlm13* (on C03) is not a result of the masking effect of A07 *Rlm* genes (*Rlm1*, *Rlm3*, *Rlm4*, *Rlm7*, and *Rlm9*). However, it is possible that another gene network is masking the allelic effect of *Rlm9*, as reported for the *AvrLm4/9*-*Rlm4/Rlm9* interaction (Ghanbarnia et al., 2018). In the present study, we could exclude this possibility, as both parental lines of a mapping population possess the *Rlm4* gene for resistance (Marcroft et al., 2012; Supplementary Table 1). Another possibility is that PHW1223 may possess an uncharacterized *Avr* gene corresponding to the *Rlm13* gene in *B. napus*.

With Illumina SNP and DArTseq markers, comparisons of physical locations of *Rlm13* and QTL detected in DH populations and diverse winter and spring GWAS panels were made in this study. We showed that the *Rlm13* is collocated with some of the QTLs for quantitative resistance that were mapped on the homoeologous regions on A03 and C03 chromosomes (Fomeju et al., 2014; Fopa Fomeju et al., 2015; Kumar et al., 2018). For example, *Rlm13* was mapped to the same genomic region associated with resistance in a diverse Australian panel of *B. napus* accessions that was assessed against multiple isolates of the *L. maculans* (D1, D2, D6, D9, and 04MGPS021), including PHW1223, used in this study (Raman et al., 2016a). It is not established whether QTL effects for resistance at the adult plant stage in described populations is either due to *Rlm13* and/or due to tight linkage between *R* and quantitative resistance locus, as suggested previously for *Rlm2* and QTL for the adult plant stage in the Darmor/Samourai population (Pilet et al., 2001; Delourme et al., 2011).

Further research is required to establish the allelic effects of *Rlm13* in diverse winter and spring ecotypes. The parents of ATR-Cobbler (ATR-Eyre and ATR-Emblem and their ancestral sources) have been extensively used in Australian canola breeding programs; as a result, several canola cultivars have been released for commercial cultivars. Our results also hint that *Rlm13* may represent a non-race-specific resistance gene that is effective against multiple isolates (described above). Similar types of resistance against multiple races of stripe rust, blast, and powdery mildew have been reported in wheat barley and rice (Buschges et al., 1997; Fu et al., 2009; Fukuoka et al., 2009). Non-race-specific resistance genes are also shown to be more durable under field conditions (Lagudah, 2011). Detection of the same consistent region for resistance to multiple isolates in the CB-Telfer/ATR-Cobbler (this study) and GWAS panel (Raman et al., 2016a) also suggests that the *Rlm13* gene is prevalent in diverse *B. napus* accessions and confers resistance to various isolates/pathotypes of *L. maculans* existing under the Australian conditions. Given that *L. maculans* is highly diverse and reproduces both sexually and asexually under field conditions, it is likely that deployment of *Rlm13* alone may not provide long-term durable resistance. Therefore, it may be deployed in different combinations with other effective *R* and QR genes to increase the level of resistance in new varieties and its durability, as demonstrated in previous studies (Brun et al., 2010). Closely linked molecular markers with *Rlm13* provide an excellent tool for various applications in Brassica improvement programs.

In conclusion, we identified a new player, the *Rlm13* gene, that is associated with resistance to blackleg in Australian canola. Based on the current and previous findings, it appears that there are two groups of qualitative *R* genes for resistance to blackleg in canola. The first group includes the race-specific resistance genes, which are effective for specific isolates of *L. maculans* such as *Rlm1*, *Rlm2*, *Rlm3*, *Rlm4*, *Rlm7*, *Rlm9*, and *Rlm10* and *LepR1-4*. The second group represents genes that provide non-race-specific resistance to multiple isolates such as *Rlm13*; however, it differs from quantitative resistance, which is described as polygenic, having small additive allelic effects and being stable over a longer evolutionary time frame (Delourme et al., 2006; Niks et al., 2015). Further research is required to verify this type of resistance in a wider *Brassica* germplasm. Nevertheless, our results provide a “target” for further understanding *Avr*–*R* gene interactions as well as provide a valuable tool for genomic-assisted breeding in canola improvement programs.

## Data Availability Statement

The original contributions presented in the study are included in the article/Supplementary Material, further inquiries can be directed to the corresponding author/s.

## Author Contributions

HR conceived the experiments, supervised the research, and drafted the manuscript. RR developed a mapping population. HR and RR conducted phenotyping experiments and genetic analysis. YQ assisted with inoculation, DNA isolation, and management of plants. JB genotyped a subset of F_2_ lines with Illumina SNP markers. YZ and SL conducted comparative mapping. All authors reviewed and approved this manuscript.

## Conflict of Interest

The authors declare that the research was conducted in the absence of any commercial or financial relationships that could be construed as a potential conflict of interest.

## Footnotes

1 http://pericles.ipaustralia.gov.au

2 www.goldenhelix.com

3 http://www.genoscope.cns.fr/brassicanapus/

4 www.aradidopsis.org
